# Supplementary material for: Dogs Can Be Reservoirs of Escherichia coli Strains Causing Urinary Tract Infection in Human Household Contacts
Source: Antibiotics (Basel). 2023 Aug 1;12(8):1269. doi: 10.3390/antibiotics12081269 (PMC10451620; doi:10.3390/antibiotics12081269)
Supplement: Supplementary file 1 [file antibiotics-12-01269-s001.zip › antibiotics-2470783-supplementary.pdf]

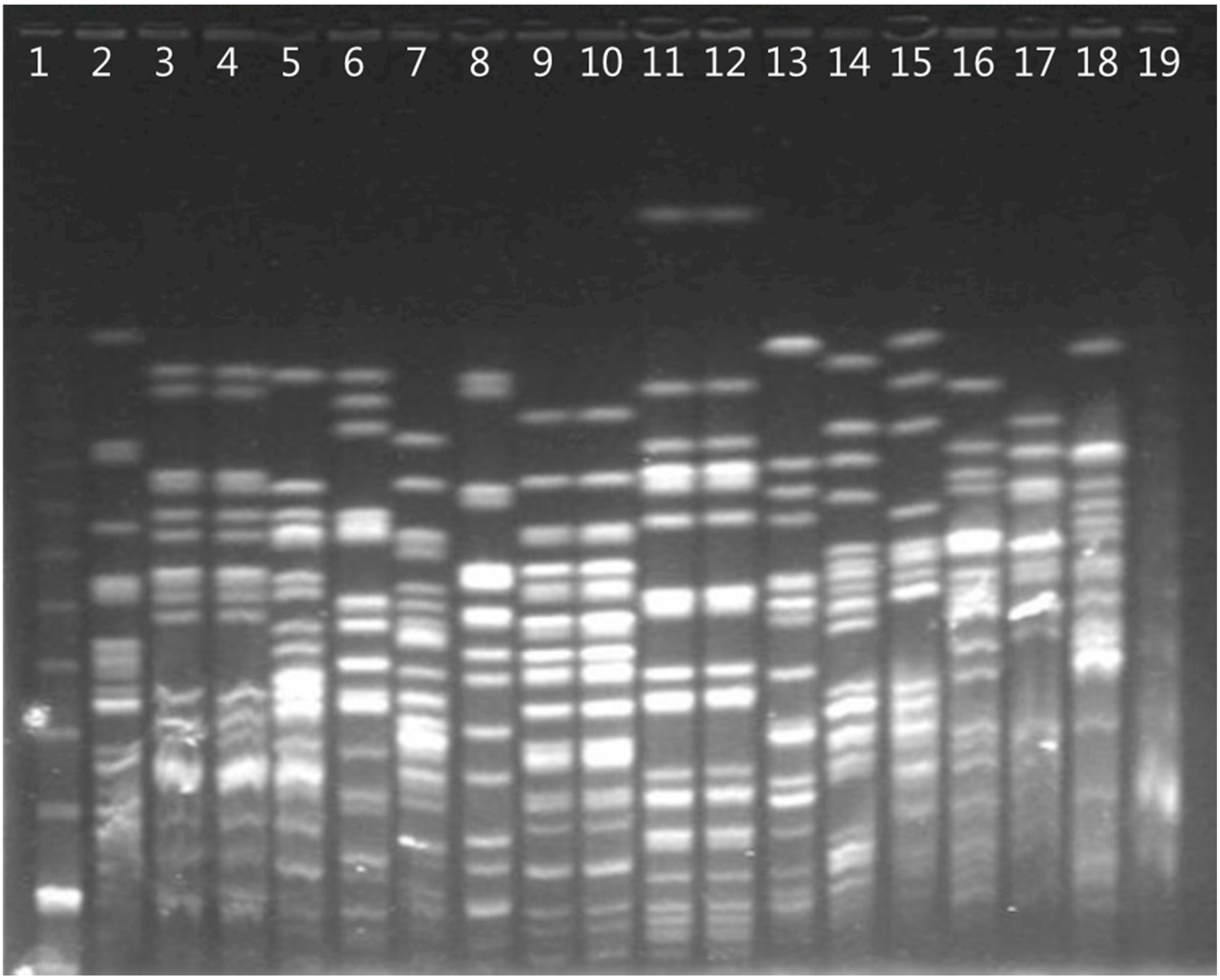

Figure S1. PFGE profiles with XbaI of *E. coli* isolates from human patients and their pets. Lanes 1 and 19, λ DNA PFGE marker; lane 2, isolate from patient of pair C; lanes 3–4, isolates from dog of pair C; lanes 5-6, isolates from pair D; lanes 7–8, isolates from dog–owner pair not described in main text (included only since dog isolate was ESBL-producer); lanes 9–10, isolates from pair A; lanes 11–12, isolates from pair B; lanes 13–14, isolates from pair E; lanes 15–16, isolates from pair F; lanes 17–18, isolates from pair G.

Table S1. Single-nucleotide polymorphism (SNP) distance matrix of strains from pair A.

	UTI	C 1st	H F-up 1	H F-up 2	H F-up 3	H F-up 4	H F-up 5	H F-up 6	C F-up 1	C F-up 2	C F-up 3	C F-up 4	C F-up 5	C F-up 6
UTI	0	2	6	5	2	7	5	2	83	86	84	83	83	83
C 1st	2	0	4	3	0	4	5	0	78	81	79	78	80	78
H F-up 1	6	4	0	5	4	0	9	4	80	83	81	80	82	80
H F-up 2	5	3	5	0	3	6	8	3	80	83	81	80	82	80
H F-up 3	2	0	4	3	0	5	5	0	79	82	80	79	81	79
H F-up 4	7	4	0	6	5	0	10	5	80	83	81	80	82	80
H F-up 5	5	5	9	8	5	10	0	5	84	88	86	85	85	85
H F-up 6	2	0	4	3	0	5	5	0	79	83	81	80	82	80
C F-up 1	83	78	80	80	79	80	84	79	0	3	1	0	2	0
C F-up 2	86	81	83	83	82	83	88	83	3	0	4	3	5	3
C F-up 3	84	79	81	81	80	81	86	81	1	4	0	1	1	1
C F-up 4	83	78	80	80	79	80	85	80	0	3	1	0	2	0
C F-up 5	83	80	82	82	81	82	85	82	2	5	1	2	0	2
C F-up 6	83	78	80	80	79	80	85	80	0	3	1	0	2	0

Abbreviations: C, canine strain; H, human strain; F-up, follow-up; UTI, strain causing UTI in the dog owner.

Table S2. Single-nucleotide polymorphism (SNP) distance matrix of strains from pair B.

	UTI	C 1st	C F-up 1	C F-up 2	C F-up 3	C F-up 4	C F-up 5	C F-up 6
UTI	0	17	29	23	28	26	40	26
C 1st	17	0	13	15	24	14	31	22
C F-up 1	29	13	0	3	7	5	24	7
C F-up 2	23	15	3	0	4	3	17	3
C F-up 3	28	24	7	4	0	59	19	4
C F-up 4	26	14	5	3	59	0	20	61
C F-up 5	40	31	24	17	19	20	0	19
C F-up 6	26	22	7	3	4	61	19	0

Abbreviations: C, canine strain; H, human strain; F-up, follow-up; UTI, strain causing UTI in the dog owner.
